# Urinary YKL-40 as a diagnostic biomarker for cystinosis

**DOI:** 10.1172/jci.insight.206193

**Published:** 2026-09-08

**Authors:** Jason H. Greenberg, Serena D Souza, Heather R. Thiessen Philbrook, Wassim Obeid, Avi Z. Rosenberg, Elena Levtchenko, Koenraad Veys, Susan L. Furth, Chirag R. Parikh

**Affiliations:** 1 Department of Pediatrics, Section of Nephrology, and; 2 Clinical and Translational Research Accelerator, Department of Medicine, Yale University School of Medicine, New Haven, Connecticut, USA.; 3 Division of Nephrology, Department of Medicine, Johns Hopkins School of Medicine, Baltimore, Maryland, USA.; 4 Department of Pathology, Division of Kidney-Urologic Pathology, Johns Hopkins University School of Medicine, Baltimore, Maryland, USA.; 5 Department of Pediatric Nephrology, Amsterdam University Medical Centers, Amsterdam, Netherlands.; 6 Department of Pediatrics, University Hospitals Leuven, Leuven, Belgium.; 7 Department of Pediatrics, The Children’s Hospital of Philadelphia, Perelman School of Medicine at the University of Pennsylvania, Philadelphia, Pennsylvania, USA.

**Keywords:** Inflammation, Nephrology, Biomarkers, Chronic kidney disease, Diagnostics

## Abstract

Early diagnosis of cystinosis is critical to limit disease progression. YKL-40, a protein in the chitinase family, released by inflammatory cells, may be a useful biomarker for cystinosis. In a case-control study of 10 children with cystinosis and 20 without cystinosis, matched by age and baseline eGFR, we measured urine YKL-40, NGAL, and EGF. A lateral flow device (LFD) for YKL-40 was also developed and tested. Urine YKL-40 was over 200-fold higher in children with cystinosis (64.6 ng/mL [IQR: 23.4, 83.8]) compared with controls (0.3 [IQR: 0.3, 0.79]; *P* = 0.0001) with excellent diagnostic discrimination (AUC = 0.99) that was superior to other biomarkers. LFD measurements for YKL-40 showed similar results (AUC = 0.93). YKL-40 results were verified in 5 cystinosis patients, and YKL-40 staining was markedly higher in kidney biopsies from cystinosis patients than in healthy controls. Urine YKL-40 has excellent diagnostic potential for cystinosis, and point-of-care technologies may facilitate early screening and management of this disease.

## Abstract 2

A new urine test may help doctors diagnose cystinosis earlier, potentially improving care for children with this rare kidney disease.

## Introduction

Nephropathic cystinosis is a rare autosomal recessive disease caused by mutations in the CTNS gene, which encodes cystinosin, a lysosomal transporter of the amino acid cystine (1). Cystinosin deficiency leads to the accumulation of intracellular and extracellular cystine crystals in the kidneys, eyes, muscles, and pancreas, resulting in inflammation and fibrosis. The accumulation of cystine crystals in the kidneys causes inflammation, tubular injury, and interstitial fibrosis, leading to progressive loss of kidney function and, in some cases, kidney failure during childhood (1).

Loss of kidney function and kidney failure in children are associated with high morbidity and mortality. Recognition of the kidney failure associated with cystinosis has allowed for a better understanding of the pathophysiology of cystinosis, development of new therapies to improve outcomes, and discovery of biomarkers that can guide clinical management and maximize the efficiency of clinical trials (2, 3). Furthermore, novel biomarkers of cystinosis may allow for the early diagnosis of cystinosis.

YKL-40 is a glycoprotein in the family of chitotriosidase enzymes produced by inflammatory cells and has a strong binding affinity for chitin. Chitin is the main component of the cell walls of fungi and protozoa, eggshells of helminths, and the exoskeletons of arthropods and insects; however, it is completely absent in mammals. YKL-40 is upregulated in kidney macrophages after ischemia-reperfusion injury and may contribute to the repair of tubular epithelium (4). Research suggests that YKL-40 may also play a key role in regulating inflammasome activation (4, 5). Cystine crystals are potent activators of human macrophages and the inflammasome system; thus, YKL-40 may serve as a good biomarker, as its levels in tissue and urine may be elevated.

In the present investigation, we assayed urine and plasma YKL-40 in patients with and without cystinosis to examine their associations with cystinosis diagnosis. We also performed YKL-40 staining in the kidneys of patients with cystinosis. We measured urine neutrophil gelatinase–associated lipocalin (NGAL) and urine epidermal growth factor (EGF) as characteristic biomarkers of tubular injury and tubular health, respectively. We hypothesized that YKL-40 levels would be elevated in urine and kidney tissue of patients with cystinosis, and high urinary YKL-40 levels would be diagnostic of cystinosis.

## Results

### Study participants.

The case-control sample consisted of 30 children: 10 with cystinosis and 20 controls matched on age and baseline eGFR (Table 1). The urine protein/creatinine (Pr/Cr) ratio was higher in those with cystinosis than in controls. The etiologies of CKD among control patients were renal dysplasia (*n* = 5), reflux nephropathy (*n* = 3), obstructive uropathy (*n* = 2), polycystic kidney disease (*n* = 1), pyelonephritis (*n* = 1), renal infarct (*n* = 1), medullary cystic disease (*n* = 1), congenital bilateral hydronephrosis (*n* = 1), and other nonspecified types of nonglomerular disease (*n* = 5).

### Correlations between urine biomarker levels, eGFR, and albuminuria.

Spearman correlations between baseline urine YKL-40 levels, urine protein/creatinine ratio, eGFR, and age are provided in Table 2. Urine YKL-40 was correlated with urine NGAL (ρ = 0.70) and urine Pr/Cr (ρ = 0.62) but did not correlate with urine EGF (ρ = 0.04) or eGFR (ρ = –0.02). The Spearman correlations comparing the YKL-40 concentration measured in urine and plasma were low (ρ = 0.15).

### Lateral flow device development.

Using ELISA results, MAB25991 and AF2599 (combination #2) were found suitable to be used as the capture antibody and detection antibody respectively (Supplemental Table 1; supplemental material available online with this article; https://doi.org/10.1172/jci.insight.206193DS1). The optimum conjugation pH was found to be 7.8 for AF2599, and an antibody-loading concentration of 15 μg/mL was found to be sufficient to form a stable gold conjugate (Supplemental Table 2). The standard curve with a lower limit of detection of 7 ng/mL was obtained using the lateral flow device (LFD) (Supplemental Figure 1). A recovery of 77% (SD 28%) and 80% (SD 21%) was obtained with a spike of 77.5 ng/mL and 15.5 ng/mL YKL-40, respectively, at a dilution of 1 in 8 (Supplemental Figure 2). This dilution was used further in the study.

### Urine biomarkers in cystinosis.

**Figure 1:**
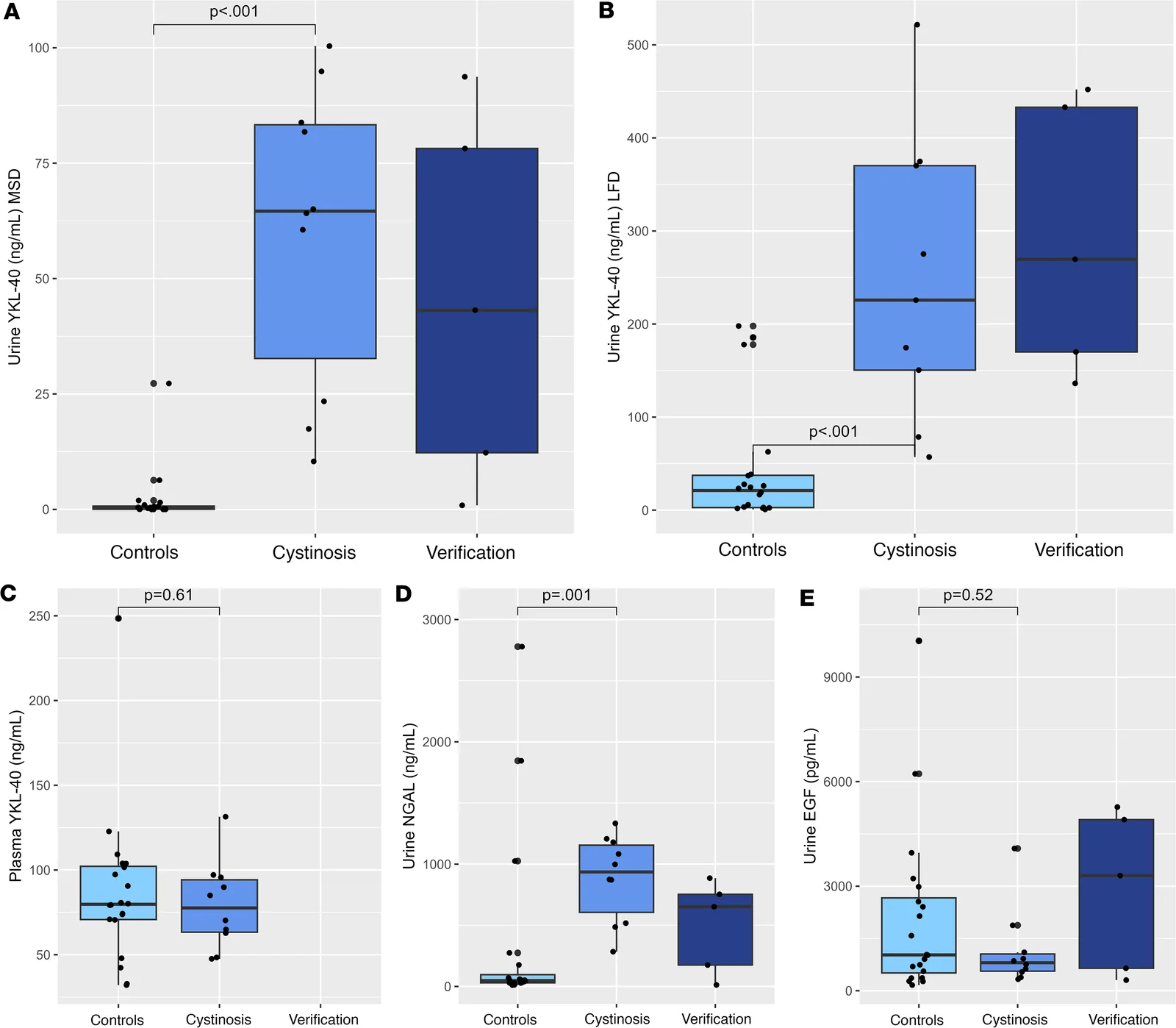
Urine YKL-40 levels in children without and with cystinosis. (**A**) Urine YKL-40 measured by MSD. (**B**) Urine YKL-40 measured by LFD. (**C**) Plasma YKL-40. (**D**) Urine NGAL. (**E**) Urine EGF.

**Figure 2:**
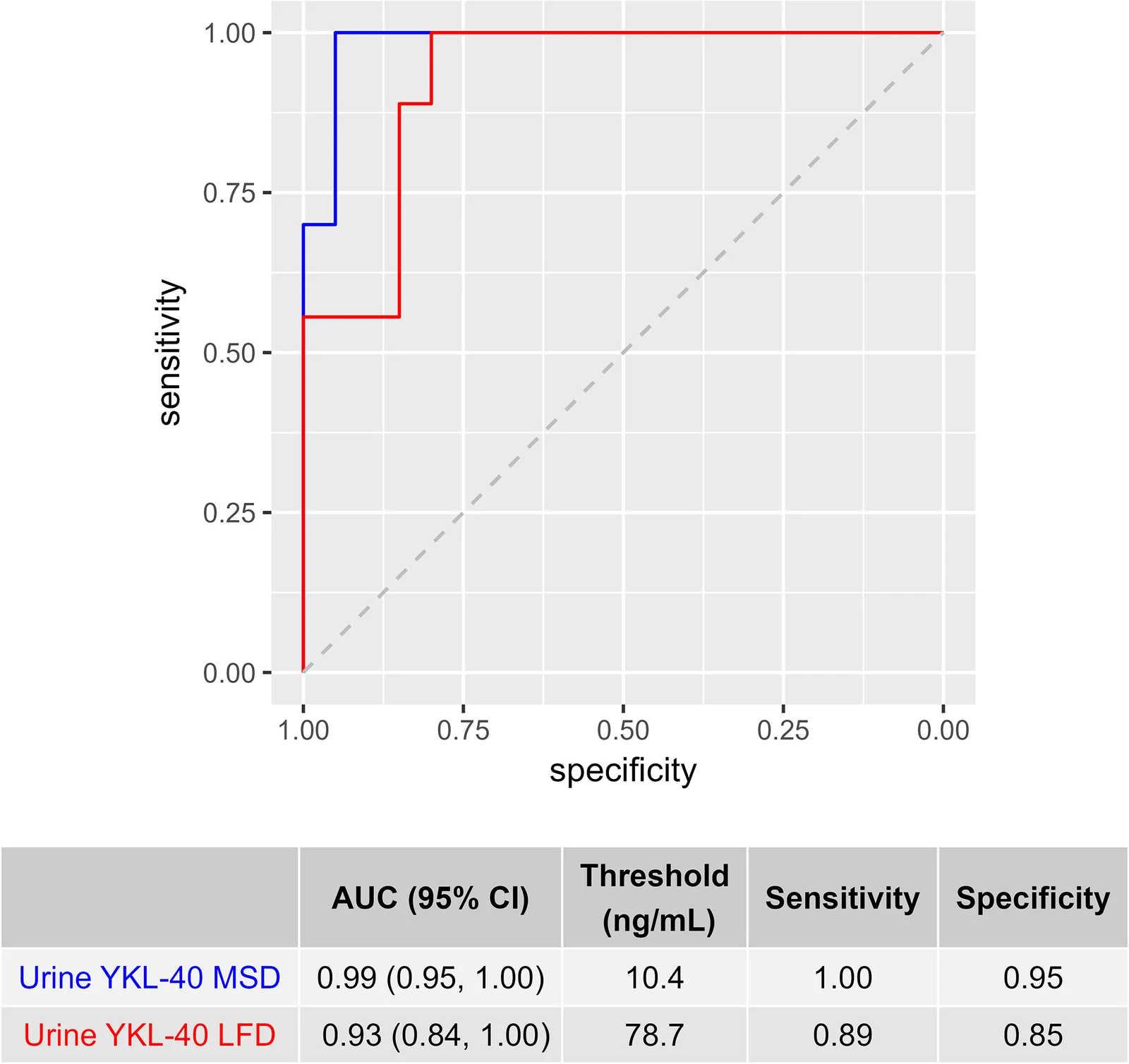
AUC for urine YKL-40 for diagnosis of cystinosis in CKiD participants.

**Figure 3:**
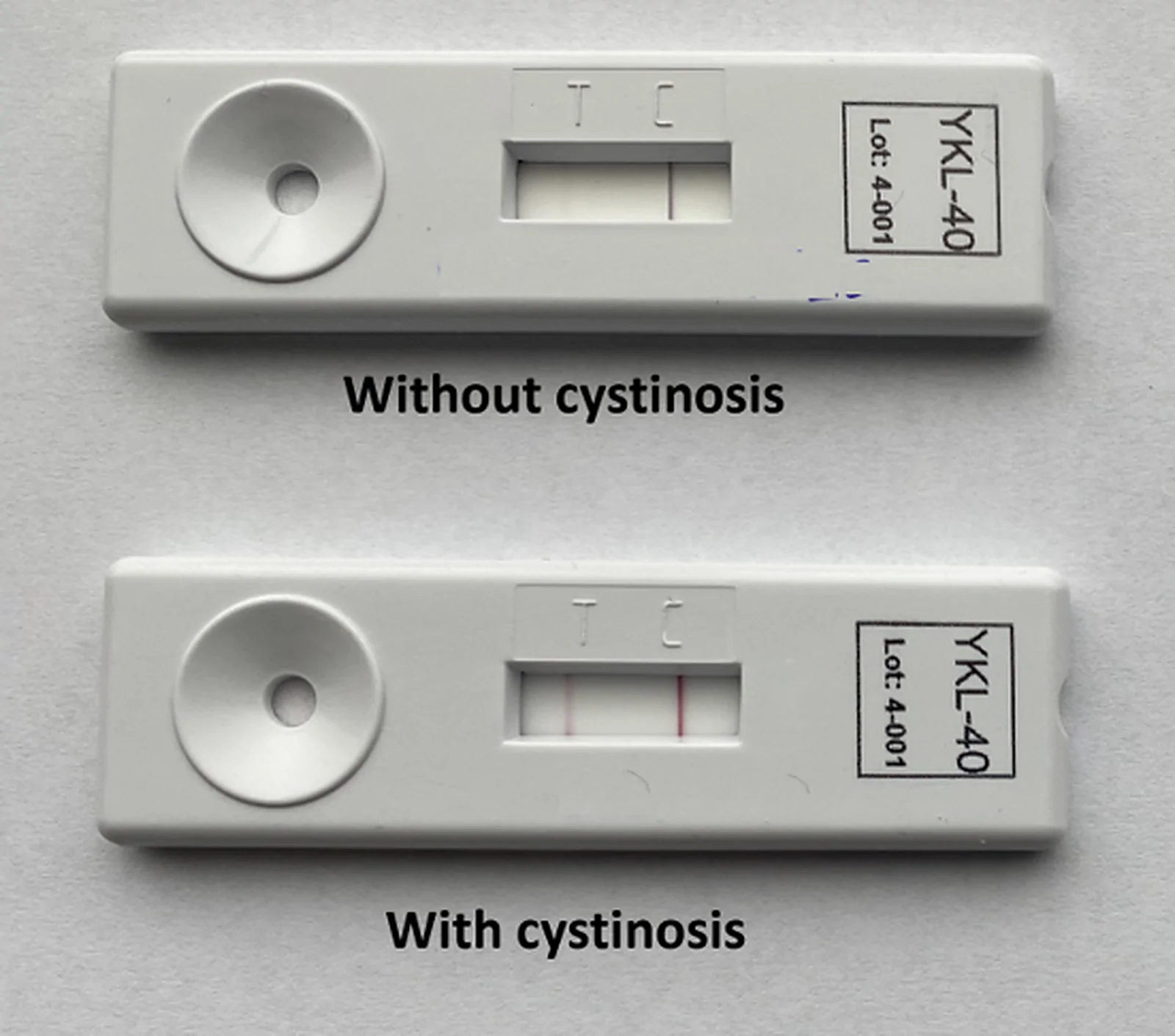
LFDs used for urine YKL-40 measurements.

In the Chronic Kidney Disease in Children (CKiD) study, individuals with cystinosis had higher levels of urine YKL-40 (64.6 ng/mL [IQR: 23.4, 83.8]) compared with controls (0.3 ng/mL [IQR: 0.3, 0.79]; *P* < 0.001), as measured by MesoScale Discovery (MSD) (Figure 1A) and by LFD (participants with cystinosis 226 ng/mL [IQR: 150, 370] versus controls 21 ng/mL IQR: [2.7, 38] *P* < 0.001; Figure 1B). In contrast, plasma YKL-40 levels were similar in the CKiD cohort in children with cystinosis and in controls (77.6 ng/mL [IQR: 62.8, 95.7] versus 79.8 ng/mL [IQR: 70.7, 102.7], respectively; *P* = 0.61) (Figure 1C). Additionally, those with cystinosis had higher levels of urine NGAL, 936.5 ng/mL [IQR: 518.8, 1179.2] as compared with controls (47.8 ng/mL ([IQR: 30.0, 123.0]; *P* = 0.001), (Figure 1D) but did not have higher levels of urine EGF than controls (*P* = 0.52) (Figure 1E). Urine YKL-40 provided excellent discrimination for the diagnosis of cystinosis (MSD measurements AUC = 0.99; LFD measurements AUC = 0.93) (Figure 2). Urine NGAL provided very good discrimination for the diagnosis of cystinosis (MSD measurements AUC = 0.87). Although there was a separation of baseline UPCR values between CKiD cases and controls (AUC = 0.95), the verification cohort of cystinosis had highly overlapping values of urine Pr/Cr compared with CKiD controls (AUC = 0.69). Urine YKL-40 > 10.4 ng/mL (measured by MSD) had a sensitivity of 100%, specificity of 95%, positive likelihood ratio of 20, and negative likelihood ratio of 0 to diagnose cystinosis, respectively. Urine YKL-40 > 78.7 ng/mL (measured by LFD) had a sensitivity of 89%, a specificity of 85%, a positive likelihood ratio of 5.93, and a negative likelihood ratio of 0.13. A representative LFD result is shown in Figure 3. Urine YKL-40 remained significantly elevated in the verification cohort in individuals with cystinosis (median 43.2 ng/mL [IQR: 12.3, 78.2]; P = 0.003) as measured by MSD and by LFD (median 270 ng/mL [IQR: 170, 433]) (*n* = 5).

### YKL-40 staining in kidney biopsy tissue.

**Figure 4:**
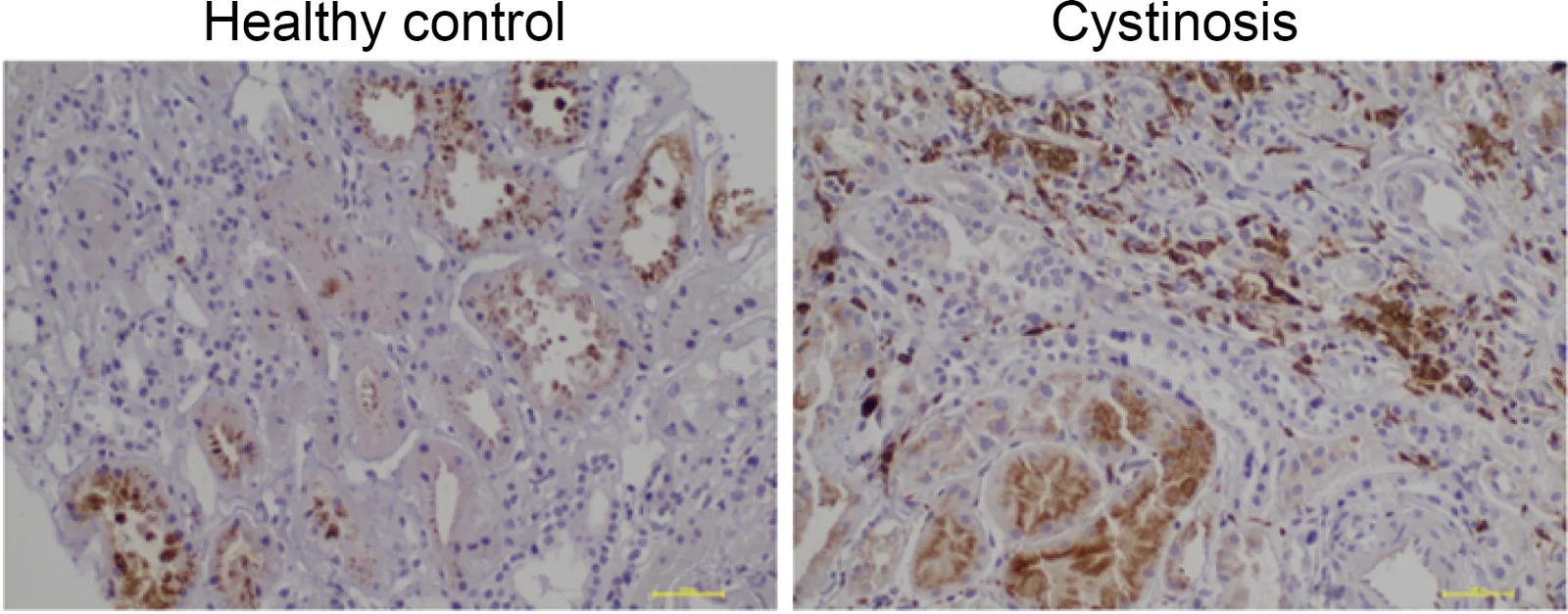
IHC staining of healthy control tissues shows no significant interstitial staining with limited background staining of tubular protein resorption droplets. By contrast in cases of cystinosis, there is a prominent interstitial staining of a cellular infiltrate. (20x magnification, 0.1 mm scale bar).

YKL-40 staining was performed on 2 cases of cystinosis and 8 healthy control tissues, the latter of which are histologically unremarkable (Figure 4). In cystinosis, staining is notable in an interstitial inflammatory infiltrate involving the cortex and medulla, focally with a peritubular distribution. Occasional podocyte staining and scattered endovascular staining were observed; however, the distribution and intensity of these findings were comparable between healthy control and cystinosis cases. Also noted is prominent tubular protein resorption droplet staining, which is similar between healthy control and cystinosis biopsies.

## Discussion

We observed that urine YKL-40 was significantly higher in children with cystinosis versus other etiologies of CKD. Additionally, we observed that urine YKL-40 remained substantially elevated in a separate cohort of children with cystinosis. Notably, urine YKL-40 levels were over 200-fold higher in those with cystinosis than in controls. While urine NGAL levels were higher in children with cystinosis, suggesting more significant tubular injury in this condition, NGAL did not reach the notable differences observed with YKL-40. This is the first study to our knowledge to describe high urine YKL-40 concentrations in children with cystinosis. To our knowledge, no other urinary biomarker has been advanced for the diagnosis of cystinosis. The high urine YKL-40 concentrations in cystinosis are consistent with profound injury and inflammation in patients with cystinosis, along with the clinical course of progressive CKD in childhood. Our observation of high YKL-40 in children with cystinosis and CKD may suggest a role for YKL-40 in the pathogenesis of cystinosis-related kidney disease. The measurement of YKL-40 using MSD is laborious, time-consuming, and requires high-end laboratory infrastructure, resulting in multiple visits and delays. This bottleneck could be addressed in the future by point-of-care approaches, including measurement of urinary YKL-40 using an LFD. In our study, LFDs demonstrated proof-of-concept feasibility with a rapid readout within 20 minutes. However, further analytical validation and standardization are needed before these assays are suitable for clinical use.

Urine YKL-40 may be high in cystinosis in response to cystine crystal accumulation in the body, particularly the kidneys. YKL-40 is highly expressed in macrophages and is likely produced as part of the inflammation associated with cystinosis. A potent inflammatory response is triggered by the cellular uptake of cystine crystals by macrophages, leading to the activation of these cells and the transcription of genes related to the inflammasome. Inflammasome-related expression of IL-1β stimulates NF-κB or induces the production of TGF-α, thus stimulating the expression of chitotriosidase. Activated macrophages and elevated chitotriosidase levels have been reported in patients with nephropathic cystinosis, supporting the role for macrophage activation as a component of disease pathophysiology (6). Additionally, YKL-40 may play a role in regulating inflammasome activation and increasing the growth rates of fibroblastic cell lines. Further research is needed to elucidate the mechanisms driving the elevations of urine YKL-40 in cystinosis.

Urine YKL-40 has emerged as a biomarker of kidney injury in a number of clinical settings. Higher urine YKL-40 concentrations have been associated with adverse outcomes including a higher risk of AKI progression, hospital mortality, and diabetic kidney disease in adults and febrile urinary tract infections in children (7–9). Our findings extend this prior body of work by demonstrating markedly elevated urine YKL-40 levels in children with cystinosis. Although urine YKL-40 is not specific to cystinosis, the magnitude of elevation observed in our study is unique and the excellent discrimination provided by YKL-40 suggests that it may have utility in cystinosis. The Hannover Reference Values for Pediatrics (HARP) study provides additional context for our present findings by describing pediatric reference intervals of urine YKL-40 during childhood (10). Although a direct comparison to the participants in this study is limited by assay and population differences, these reference ranges highlight the markedly high levels of urine YKL-40 observed in cystinosis.

The clinical value of a sensitive, accessible test for cystinosis derives from the narrow therapeutic window of the disease. Cystine accumulation and proximal tubular injury begin in infancy, and early initiation of cysteamine is a principal determinant of long-term kidney survival, so diagnostic delay translates directly into irreversible nephron loss (11). However, leukocyte cystine quantification by liquid chromatography-tandem mass spectrometry, the diagnostic standard, is available only at specialized centers and requires cold-chain transport and immediate processing of intact cells. In milder or juvenile disease, leukocyte cystine may also fall below diagnostic cut-offs and yield false-negative results, while plasma chitotriosidase is uninformative in individuals homozygous for the *CHIT1*-null variant (12).

Elevated urine YKL-40 in children with cystinosis may have implications for the diagnosis of cystinosis and novel therapeutic approaches. Urine YKL-40 may serve as a biomarker for the diagnosis of cystinosis. Urine YKL-40 levels can be measured using immunoassays, which can be set up in most hospital laboratories. Furthermore, with additional development, LFDs could enable more rapid, decentralized testing closer to the point of care, with less operator training and potential applicability in resource-limited settings. The availability of an LFD in the future would also greatly enhance screening for children for this condition, since those who test positive on LFD could have their results confirmed with more definitive immunoassays. Positioned this way, it functions as a sensitive first-tier screen that flags children for confirmatory leukocyte cystine or *CTNS* testing rather than as a stand-alone diagnostic. At present, additional work is required on our YKL-40 LFD to establish its diagnostic performance, reproducibility, and validity before it can be incorporated into routine clinical workflows.

Although urinary YKL-40 concentrations measured by the LFD were higher than those obtained using the MSD assay, absolute biomarker values were not expected to be directly comparable across assay platforms. Differences in antibody characteristics, analytic sensitivity, and susceptibility to urine matrix effects can all affect urine YKL-40 concentrations. Importantly, the objective of the LFD was to establish a proof of concept for rapid point-of-care detection rather than an equivalent test to the laboratory-based MSD assay. Accordingly, the preservation of discrimination between cystinosis and control samples was our priority rather than the measured concentrations. Additional assay optimization and formal validation will be required before clinical implementation.

Prior work in the CKiD cohort demonstrated that urine YKL-40 is also elevated in children with glomerular CKD, supporting that glomerular injury may contribute to higher urinary YKL-40 levels (13). However, the magnitude of elevation observed in cystinosis in the current study (>200-fold relative to nonglomerular CKD controls) suggests that glomerular injury alone is unlikely to account for this signal. We selected nonglomerular CKD controls to reduce confounding from glomerular injury. Given that cystinosis is characterized by proximal tubular dysfunction, this design was intended to focus on a tubular injury related biomarker signal. However, cystinosis is not purely a tubular disorder, and progressive disease may include glomerular involvement. Therefore, elevations in urine YKL-40 in cystinosis patients may reflect a combination of tubular injury and glomerular pathology. Accordingly, the extent to which urine YKL-40 distinguishes cystinosis-specific injury from broader glomerular and Fanconi-related pathology remains uncertain. An additional limitation is that the verification cohort included only children with cystinosis and did not include a control group. Future studies with a broader control group including alternative causes of Fanconi syndrome will be important to delineate the specificity of urine YKL-40 and to determine whether it provides additional diagnostic value.

There are other limitations to currently available tests. White blood cell cystine levels require tandem mass spectrometry and need standardized reference values due to variations based on leukocyte populations and methodologies (14). Thus, plasma chitotriosidase, an enzyme produced by activated macrophages, is suggested for long-term therapeutic monitoring of nephropathic cystinosis, as it has several advantages over white blood cell cystine levels (15–17). YKL-40 and chitotriosidase are both linked through the chitinase family of enzymes, and YKL-40 levels in the urine may have utility as a noninvasive substitute for plasma chitotriosidase for therapeutic monitoring.

Urine YKL-40 may not only serve as an early biomarker of kidney disease in cystinosis, allowing for early intervention to limit the loss of kidney function; measuring YKL-40 over time may also indicate disease severity and enable clinicians to monitor the response to treatment. Advances in newborn screening programs have enabled early detection and intervention for several genetic disorders (18). The *CTNS* gene harbors a diverse range of pathogenic variants associated with cystinosis, and the distribution and frequency of these variants in different populations make the development of a targeted genetic screening program challenging. A urine biomarker like YKL-40 would allow for a population-based screening test for early diagnosis. A global survey revealed significant disparities in access to cysteamine treatment and diagnostic investigations for neonatal cystinosis, particularly in developing countries, and LFDs for YKL-40 could be deployed globally as they are relatively inexpensive and easy to use (19).

Lastly, given YKL-40’s role in inflammation and fibrosis, it may represent a therapeutic target in children with cystinosis. Modulating YKL-40 expression or activity could reduce inflammation and tissue injury. Further research on YKL-40 and its role in inflammasome activation may identify novel therapeutic approaches to target YKL-40 or other proteins in its pathway.

## Methods

### Sex as a biological variable

Both female and male participants were included in the discovery and verification cohorts. Sex was not evaluated as a biological variable because the sample size was not large enough to evaluate sex-specific differences. Cystinosis affects both males and females and the findings are expected to be relevant to both sexes.

### Study participants

#### Derivation cohort.

Cases and controls were identified from the CKiD study. The CKiD study is a cohort study of children with CKD enrolled from 54 medical centers in the United States and Canada from 2006 through 2016 (20, 21). Children were prospectively enrolled in the CKiD study if they were between 6 months and 16 years old and had an eGFR of 30–90 mL/min/1.73 m^2^. Children were excluded if they had a history of kidney, solid-organ, or bone marrow transplantation; dialysis within 3 years; or a history of cancer. CKiD participants underwent annual study visits to assess their height, weight, medication use, blood pressure, eGFR, and urine albumin levels.

Children with a diagnosis of cystinosis were included in the study as cases if they had sufficient volume of stored urine and had data on kidney function (eGFR and urine albumin/creatinine ratio). We matched each case with cystinosis based on age and baseline eGFR with 2 CKiD participants without cystinosis and with nonglomerular etiologies of CKD (most commonly obstructive uropathy, kidney dysplasia, or reflux nephropathy).

#### Verification cohort.

To verify our results, urine was obtained from individuals with cystinosis in a Belgium cystinosis clinic at the University Hospital Leuven, Belgium. Demographic and clinical data were collected from participants.

### Clinical and laboratory variables

We determined the eGFR using published equations derived from the CKiD population based on serum creatinine, cystatin C, and blood urea nitrogen concentrations (22). eGFR was measured at the baseline study visit in the CKiD and Belgium cohorts. Body mass index was standardized for age and sex. Serum creatinine and eGFR were measured annually in CKiD. Serum creatinine measurements were performed in the same CKiD central laboratory at the University of Rochester. Hypertension was defined as a systolic or diastolic blood pressure ≥ 95th percentile for age, sex, and height or ≥ 130/80 mmHg, whichever blood pressure threshold is lower (23).

### Biomarker measurements

Stored biospecimens were centrifuged at 1,100–1,300*g*, after which the urine supernatant was aliquoted. Barcoded urine aliquots were stored at –80°C until biomarker measurement.

#### Meso scale discovery (MSD) measurements.

Urine YKL-40, NGAL, and EGF were measured in duplicate using an MSD electrochemiluminescence-based multiplex assay (MSD). The mean values for each biomarker were used in the analyses. Biomarker measurements were repeated on participants’ urine samples if 2 or more analytes had intraassay coefficients of variation (CVs) > 15%. The intra- and interassay CVs were all < 10%. Plasma YKL-40 concentrations were previously measured in CKiD participants using an MSD multiplex assay as previously described (24), and these plasma results were obtained to study the correlation between plasma and urine YKL-40.

### LFD development and measurements

The details for the development of LFD are provided in the Supplemental Methods. Briefly, MAB25991 and AF2599 (combination #2) were selected as the capture and detection antibodies following ELISA-based screening (Supplemental Table 1 and Supplemental Methods). The conjugation buffer pH and loading concentration of the antibody was optimized for gold nanoparticle conjugation. These antibodies were further used for the LFD development (Global Access Diagnostics) and tested using recombinant protein (2599-CH-050) to obtain a concentration-dependent standard curve. Optimal dilution of urine to be used was determined from spike and recovery experiments.

### Measurement of urinary YKL-40 in cohort samples using LFDs

From participants with available stored samples, all cases (*n* = 14; 9 CKiD, 5 non-CKiD) and controls (*n* = 20) had urine YKL-40 measured in duplicate using an LFD. For the measurement, the centrifuged urine samples were diluted (1:8) and added to the custom-made diluent tube. The diluent tube was inverted 5 times. Three drops of the diluted sample were added to the LFD and incubated for 20 minutes. The results were read using a handheld RDS-2500 reader (DETEKT) in the form of a ratio of test line (TL) to control line (CL) intensity. The ratio was then converted to the corresponding concentration using a standard curve of recombinant protein. The analytical precision of the urine YKL-40 LFD was evaluated using replicate measurements of 3 participant urine samples. The interassay CV was 7%.

### IHC for kidney biopsy tissue

Patient-derived samples were used from the Johns Hopkins Renal Pathology archives under an IRB-approved protocol. Formalin-fixed and paraffin-embedded human tissue slides were deparaffinized with xylene and rehydrated with ethanol, and heat-induced epitope retrieval was performed in a pressure cooker with citrate buffer (BioSB Inc, BSB 0022). Following peroxidase quenching (Bio SB Inc., BSB 0054), the slides were incubated with rabbit monoclonal anti–YKL-40 antibody (1:800) (Cell Signaling, 47066) for 90 minutes with detection using high sensitivity Mouse/Rabbit PolyDetector Plus DAB HRP Brown Detection System (Bio SB Inc., BSB 0269) and hematoxylin counterstain.

### Statistics

The primary exposure was assessed by measuring urine YKL-40, NGAL, and EGF concentrations from stored urine samples. The urine specimens were collected a median of 5 months (IQR: 4–7) after the baseline visit in CKiD and at study enrollment in the Belgium cohort. The urine YKL-40, NGAL, and EGF concentrations are presented in units of ng/mL. The primary outcome was the diagnosis of cystinosis. Cystinosis is diagnosed clinically at the participant’s local site by detecting elevated cystine content in peripheral blood leukocytes or cystine corneal crystals, or identifying pathologic variants of the cystinosin gene. The Spearman correlations between the biomarkers, age, eGFR, and urine Pr/Cr ratio were estimated (25). We also estimated the Spearman correlation between urine and plasma YKL-40. For the primary analysis of the case-control study, we compared levels of urine YKL-40, NGAL, and EGF between individuals with and without a diagnosis of cystinosis with the Mann-Whitney *U* test. To evaluate the diagnostic value of urine YKL-40 in cystinosis, we determined the area under the receiver operating characteristic curve (AUC) for each biomarker measurement method separately (MSD and LFD). In the verification sample, we determined the levels of urine YKL-40 in an additional group of patients with a diagnosis of cystinosis. Analyses were performed using SAS 9.4 for Windows (SAS Institute Inc., NC) and R (R Core Team Version 3.5.1).

### Study approval

The CKiD and Belgium studies were approved by the IRB of each participating institution.

### Data availability

Values for all data points in graphs are reported in the Supporting Data Values file.

## Author contributions

JHG and CRP conceived and designed the study. JHG supervised the study, acquired clinical data, interpreted the findings, and wrote the first draft of the manuscript. SD and WO designed, optimized, and validated the lateral flow assay and performed the laboratory experiments. HRTP performed the statistical analyses and contributed to interpretation of the data. AZR performed and interpreted the immunohistochemical analyses. EL and KV assembled the verification cohort and contributed to interpretation of the findings. SLF contributed to study design and interpretation of the data. All authors contributed to manuscript revision, approved the final version, and accept responsibility for the integrity of the work.

## Conflict of interest

CRP declares consulting arrangement with Alexion and Astra Zeneca pharmaceuticals.

## Funding support

This work is the result of NIH funding, in whole or in part, and is subject to the NIH Public Access Policy. Through acceptance of this federal funding, the NIH has been given a right to make the work publicly available in PubMed Central.

- CKD Biomarkers Consortium (National Institute of Diabetes and Digestive and Kidney Diseases grants U01 DK085689, U01 DK102730, U01 DK103225, U01 DK085660) to SLF and CRP.
- NIH grants K08DK110536, R01DK135650, and R01DK135518 to JHG.
- NIH grants U54DK137331, U01DK114866, U01DK129984 and R01DK093770 to CRP.
- NIH K24DK078737 and U01DK66174 to SLF.
- Edward S. Kraus grant 2023-2024 to SD
- CKiD (U01-DK-66143, U01-DK-66174, U01DK-082194, U01-DK-66116).

## Supplementary Material

Supplemental data

ICMJE disclosure forms

Supporting data values

## Figures and Tables

**Table 1:**
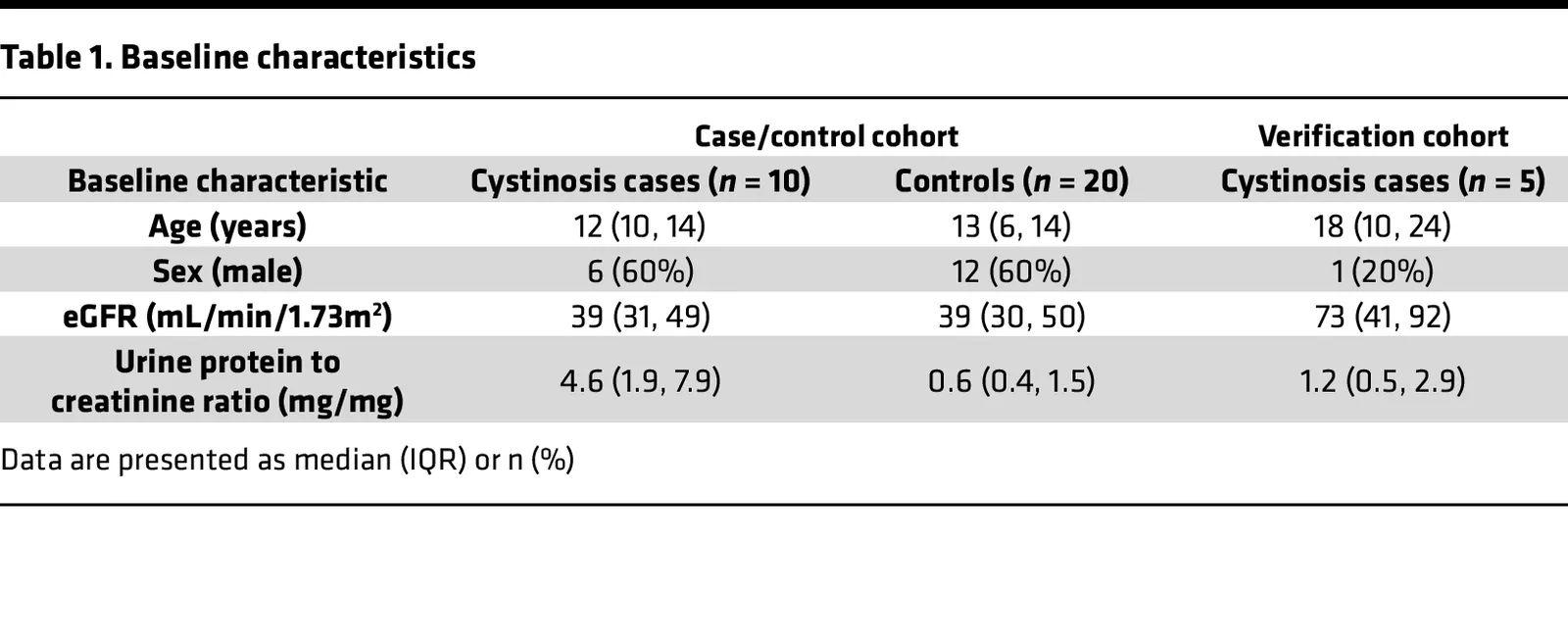
Baseline characteristics

**Table 2:**
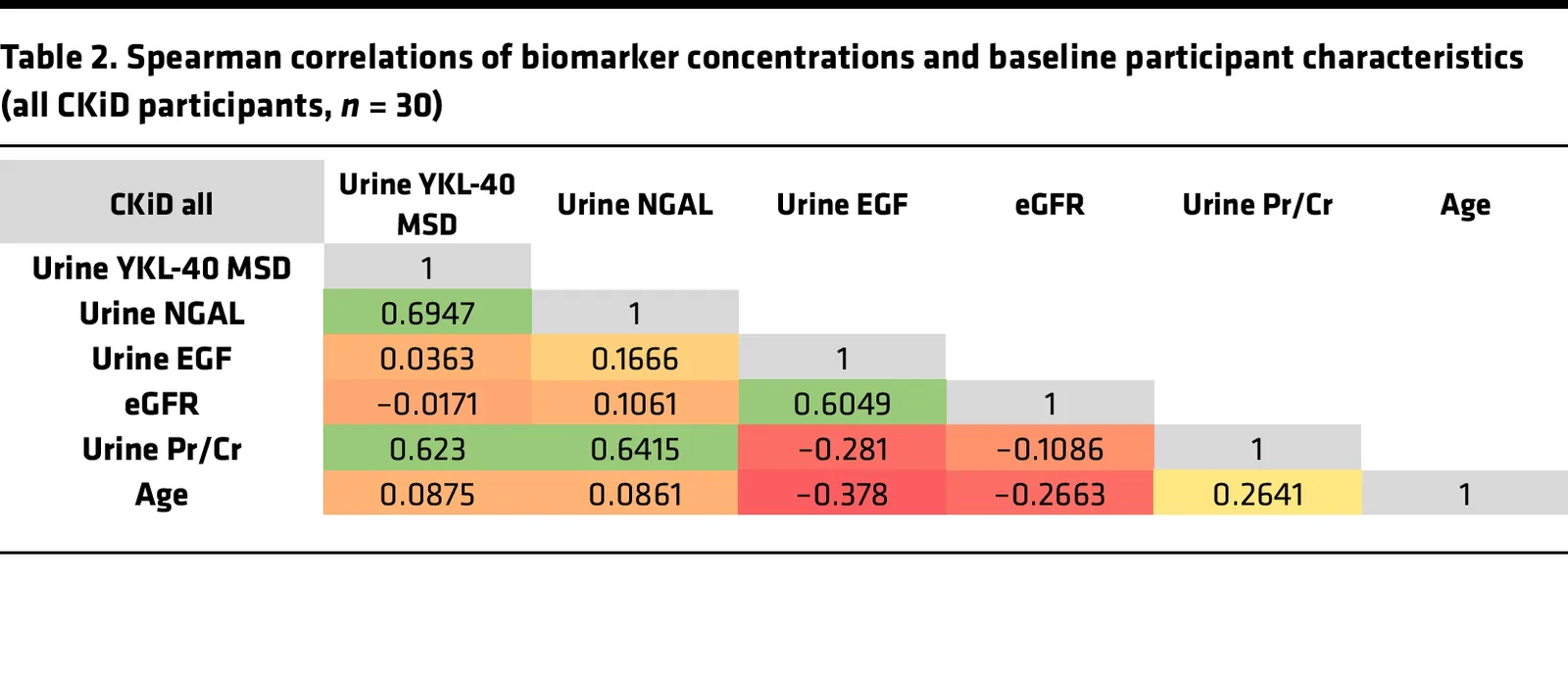
Spearman correlations of biomarker concentrations and baseline participant characteristics (all CKiD participants, *n* = 30)

## Version 1 09/08/2026

Electronic publication
