# Supplementary material for: Urinary YKL-40 as a diagnostic biomarker for cystinosis
Source: JCI Insight. 2026 Sep 8;11(17):e206193. doi: 10.1172/jci.insight.206193 (PMC13564077; doi:10.1172/jci.insight.206193)
Supplement: Supplementary file 1 [file jciinsight-11-206193-s074.pdf]

SUPPLEMENTAL MATERIAL

Urinary YKL-40 as a Diagnostic Biomarker for Cystinosis

Jason H. Greenberg,[1,2] Serena D Souza,[3] Heather R. Thiessen Philbrook,[3] Wassim Obeid,[3] Avi Z. Rosenberg,[4] Elena Levtchenko,[5] Koenraad Veys,[6] Susan L. Furth,[7] and Chirag R. Parikh[3]

[1]Department of Pediatrics, Section of Nephrology, and [2]Clinical and Translational Research Accelerator, Department of Medicine, Yale University School of Medicine, New Haven, Connecticut, USA.
[3]Division of Nephrology, Department of Medicine, Johns Hopkins School of Medicine, Baltimore, Maryland, USA.
[4]Department of Pathology, Division of Kidney-Urologic Nephrology, Johns Hopkins University School of Medicine, Baltimore, Maryland, USA.
[5]Department of Pediatric Nephrology, Amsterdam University Medical Centers, Amsterdam, Netherlands.
[6]Department of Pediatrics, University Hospitals Leuven, Leuven, Belgium.
[7]Department of Pediatrics, The Children's Hospital of Philadelphia, Perelman School of Medicine at the University of Pennsylvania, Philadelphia, Pennsylvania, USA

Contents

Supplementary Methods 2
Supplementary Table 1: Immunological testing of antibodies for YKL-40 4
Supplementary Table 2: Conjugation buffer pH and loading concentration optimization 5
Supplementary Figure 1: Recombinant protein standard curve for YKL-40 using lateral flow device 6
Supplementary Figure 2: Spike and recovery at (A) 77.5 ng/mL and (B) 15.5 ng/mL of YKL-40 7

Supplementary Methods

Immunological testing of the antibody using ELISA

The microtiter ELISA plates (Costar 9018, Thermo Fisher Scientific) were coated with 100 µL of antibody (AF2599/MAB25991) at a concentration of 1 µg/mL in PBS and incubated overnight at room temperature. The plates were washed thrice with 300 µL TBST using a plate washer (BioTek 405). The wells were blocked with 150 µL PBST 0.1 % BSA 1% and incubated for 1h at room temperature. The blocking solution was discarded, the plates were washed thrice with 300 µL TBST and incubated at room temperature with respective 100 µL antigen for 1h on shaker. After discarding the antigen solution, the plates were washed thrice with 300 µL TBST and incubated with the respective 100 µL antibody (AF2599/MAB25991;1 µg/mL in PBST 0.1 % BSA 1%) for 1h at room temperature on shaker. Post incubation, the antibody solution was discarded and following a final wash, the plates were incubated at room temperature with 100 µL of respective anti-species alkaline phosphatase at a dilution of 1: 10k for 1h on shaker at room temperature. The plates were incubated at room temperature in the dark with 100 µL pNPP and the absorbance was read at 405 nm after 30 min.

Conjugation buffer pH optimization and conjugation concentration optimization

1.5 µL of 1 mg/mL detection antibody was mixed with 5 µL of conjugation buffer (pH spanning from 5.3 to 9.3 - 20mM MES pH 5.3, 20mM BES pH6.4, 20mM TES pH7.1 & 7.5, 20mM TAPS pH 7.8 & 8.5, 20mM Borate pH 9.0 & 9.3) and 0.1 mL colloidal gold (EM.GC40, BBI Solutions) in microtiter ELISA wells (StarLab, E2996-1600) on a plate shaker for 10 min. The absorbance was read at 530 nm, 550 nm and 600 nm on FLUOstar Omega Microplate Reader (BMG Labtech) to calculate the aggregation ratio of Abs 550/600. Antibody loading concentration was determined by preparing a loading concentration of 0-30 µg/mL by mixing 5 µL of the selected buffer with varying volumes of antibody solution (1 mg/mL). 100 µL of colloidal gold was added to the mixture and incubated for 10 minutes. The mixture was subjected to a salt challenge by adding 10 µL of 1M NaCl. The absorbance was read at 530, 550 and 600 nm on FLUOstar Omega Microplate Reader (BMG Labtech). The aggregation ratio of Abs 550/600 was calculated.

Lateral flow strip development

The lateral flow test strip (Global Access Diagnostics, USA) consisted of a sample pad/GC pad (Ahlstrom-Munksjo, USA, Product 8980), CN180 nitrocellulose test membrane (Sartorius, USA) and a wicking adsorbent pad (Ahlstrom-Munksjo, USA, Product 222) onto a backing card (Kenosha, Netherlands, product KN-V1060-221). The test line and control line were prepared using anti- human YKL-40 MAB25991 (1 mg/mL, R&D systems, USA) and Rabbit anti-goat IgG (1 mg/mL, Jackson Immunoresearch, USA, Product code 305-005-003) respectively. The membranes were dried at a temperature of 55°C for 2 minutes. The LFD was assembled and stored until further use.

Lateral flow device testing using recombinant protein standards

A recombinant protein standard curve was generated for YKL-40 (0-1000 ng/mL) using the lateral flow device. Three drops of the YKL-40 standard (prepared and filled in a custom-made diluent tube) were added to the device and incubated for 20 minutes on a flat surface. The results were read using a handheld RDS-2500 reader (DETEKT, USA) in the form of control (CL) and test line (TL) intensity. A ratio of the TL/CL intensity was computed to analyze the results. The spike and recovery were determined by using urine samples from deceased donors (IRB IRB00248332). The urine samples were spiked with recombinant protein standard at 77.5 ng/mL and 15.5 ng/mL in diluted urine samples (1:2, 1:4 and 1 in 8).

Supplementary Table 1: Immunological testing of antibodies for YKL-40

YKL-40		
Standard (pg/mL)	Combination #1<br><br>AF2599 (Capture Ab) MAB25991 (Detection Ab)	Combination #2<br><br>MAB25991 (Capture Ab) AF2599 (Detection Ab)
	Mean Absorbance (SD)	
25000	0.41 (0.01)	3.09 (0.07)
5000	0.10 (0.00)	1.40 (0.04)
1000	0.08 (0.00)	0.29 (0.01)
200	0.07 (0.00)	0.11 (0.00)
40	0.08 (0.01)	0.09 (0.01)
8	0.07 (0.00)	0.09 (0.00)
1.6	0.07 (0.00)	0.1 (0.00)
0	0.07 (0.01)	0.09 (0.00)

Combination #2 was found to be optimal for the study

Supplementary Table 2: Conjugation buffer pH and loading concentration optimization

Conjugation buffer pH	AF2599 (Detection antibody) for YKL-40		Antibody loading concentration (µg/mL)	AF2599 (Detection antibody) for YKL-40
	**Absorbance ratio (550/600)**			**Absorbance ratio (550/600)**
9.3	4.00		**0**	1.3
**9**	4.14		**5**	**1.0**
**8.5**	4.29		**10**	**1.8**
**7.8**	4.29		**15**	**4.3**
**7.5**	4.31		**20**	**4.2**
**7.1**	4.04		**25**	**4.3**
**6.4**	3.19		**30**	**4.3**
**5.3**	0.97			

Supplementary Figure 1: Recombinant protein standard curve for YKL-40 using lateral flow device

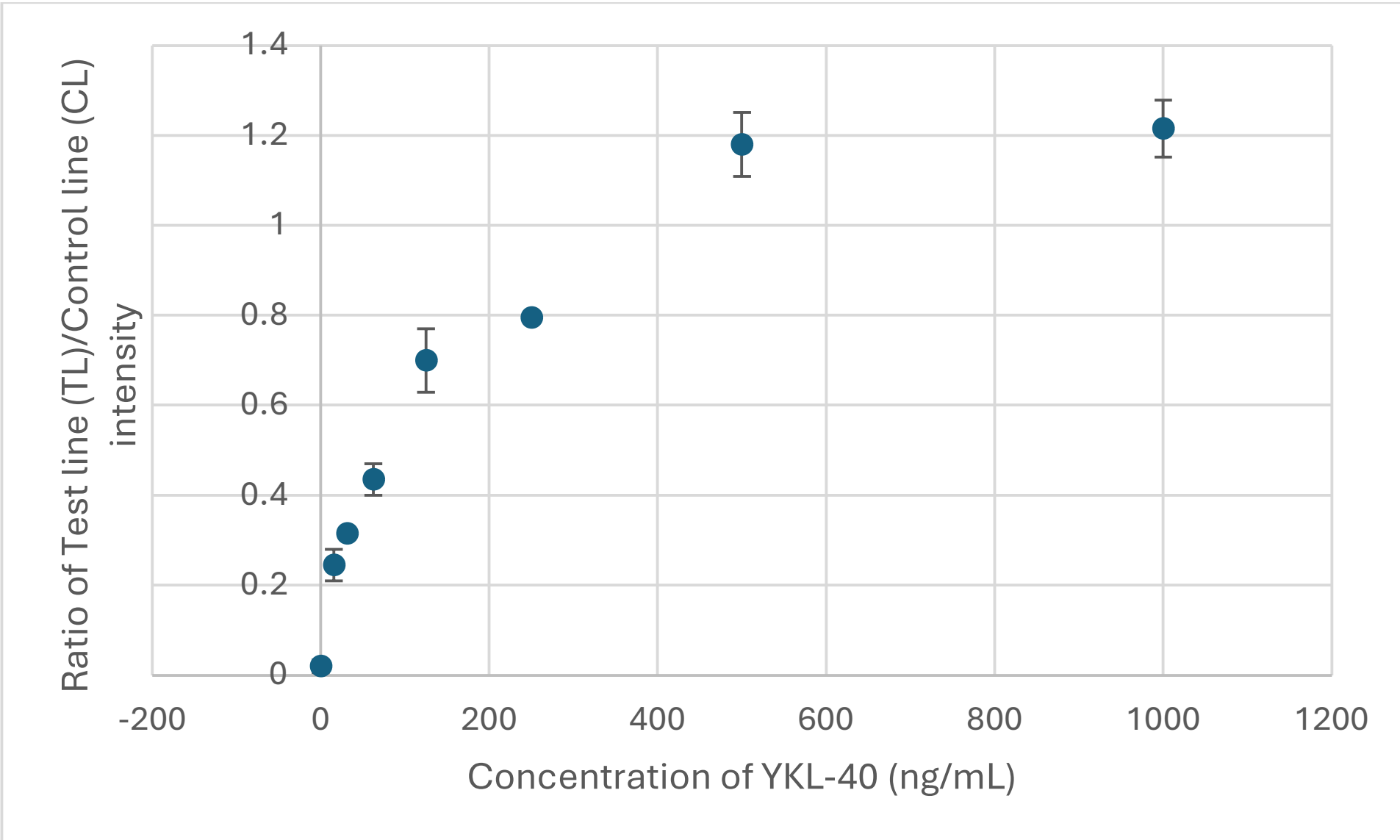

Supplementary Figure 2: Spike and recovery at (A) 77.5 ng/mL and (B) 15.5 ng/mL of YKL-40

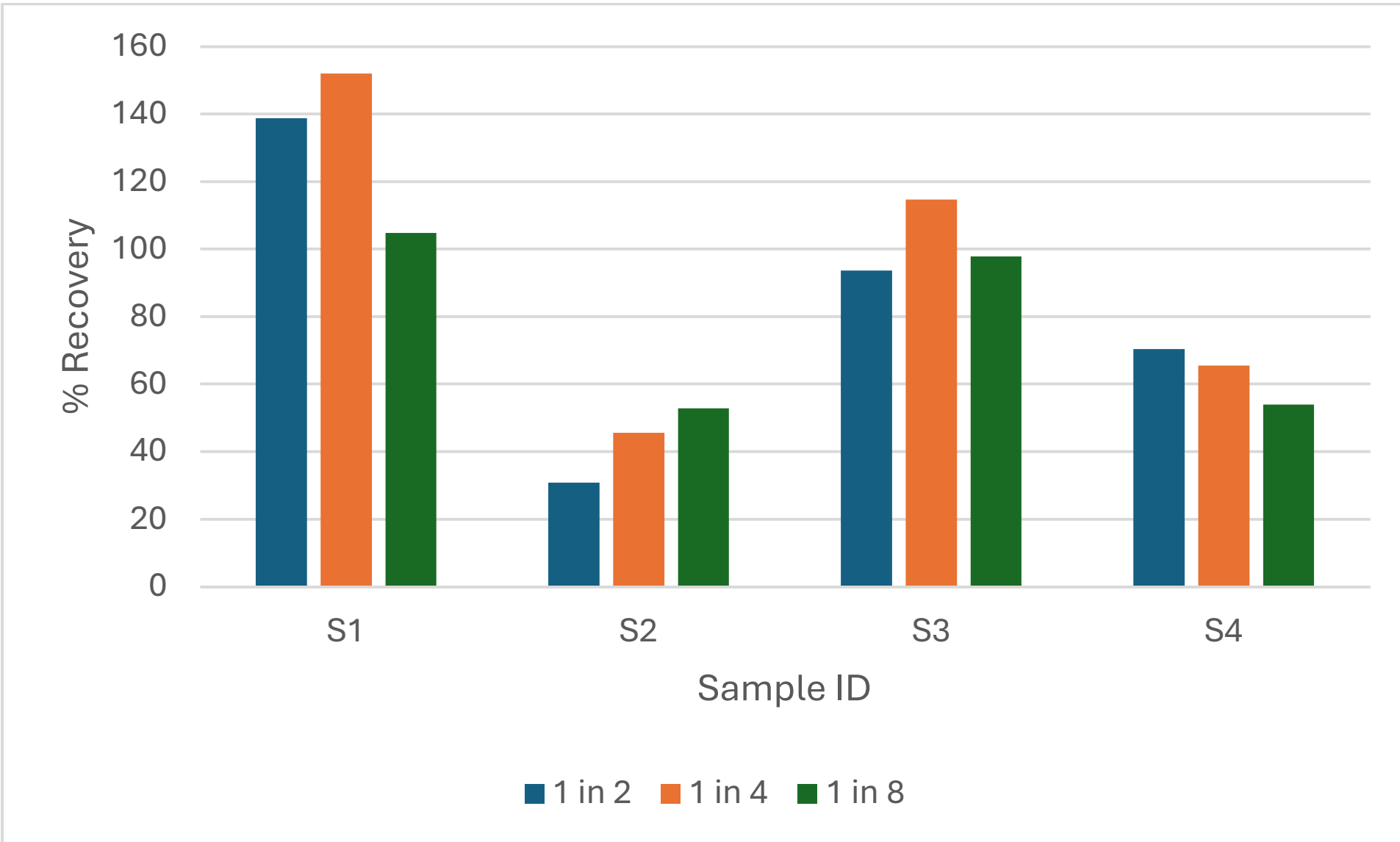

A

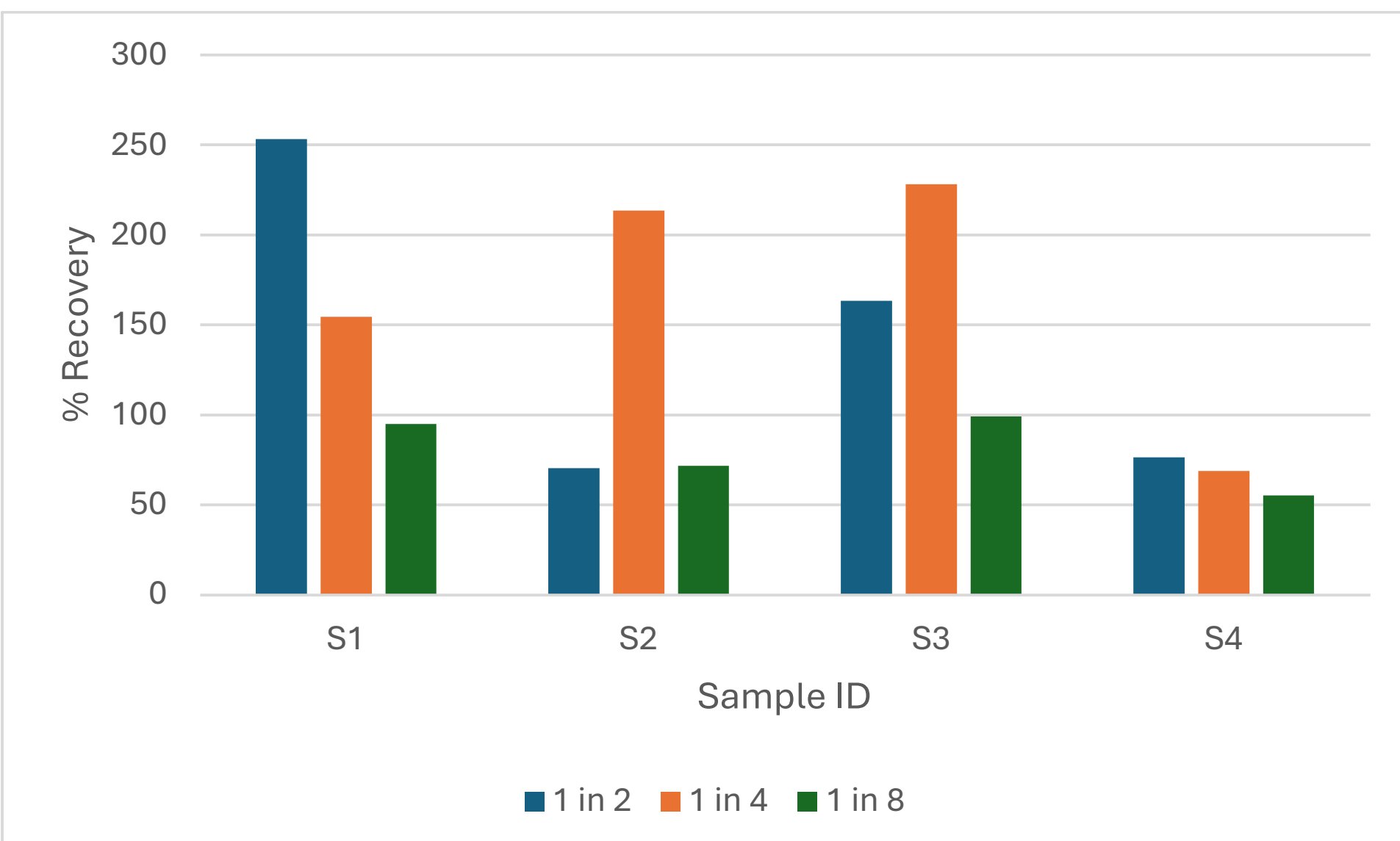

B
